# Critical Walking Methodologies and Oblique Agitations of Place

**DOI:** 10.1177/10778004211042355

**Published:** 2021-09-02

**Authors:** Stephanie Springgay, Sarah E. Truman

**Affiliations:** 1 McMaster University, Hamilton, Ontario, Canada; 2 The University of Melbourne, Victoria, Australia

**Keywords:** critical walking methodologies, place, oblique relations and contours

## Abstract

In this Editorial, we discuss *WalkingLab*’s approach to critical walking methodologies grounded in queer-feminist, anti-racist praxis, and argue for the need to critically account for understandings of place in times of ongoing crises. We then introduce the articles featured in this special issue. Authored by international scholars, each article in the special issue engages critically with walking methodologies and the concept place from oblique angles.

Walking has been socially, ethically, and politically imbricated in the world’s response to ongoing global crises including the COVID-19 pandemic, climate change, anti-Black racism, and Indigenous genocide. As countless places have entered various stages of lockdown over the past 2 years, the capacity to walk has taken on new meanings. While walking afforded some people the ability to exercise, to move outside of their homes, or provided an opportunity to socialize in a distanced way, for other people walking was heavily policed or risky in dense urban spaces, and for others the opportunity to walk was removed altogether and there were reports of long-term care facilities locking residents in their rooms, entire apartment blocks placed under police guard in Melbourne, and people with disabilities losing the necessary attendant care to walk interdependently. The pandemic has further exacerbated walking’s inequities.

Siddique Motala and Vivienne Bozalek (this issue) argue that walking is further complicated by its geographic specificity, and as such walking methodologies in the Global North do not easily translate to the Global South (nor should it). One might argue that this is precisely why a more robust interrogation of walking and place is urgently needed to take into account not only intersectional frameworks but also the ways that place is socially, materially, and politically entangled with walking. This special issue on critical walking methodologies pays careful attention to the specificities of place and the ways that place is imbricated in ongoing global crises. For *WalkingLab*, critical walking methodologies insist that intersectionality, the place where research takes place, and how one moves through space be critically complicated and accounted for (Springgay & Truman, 2018). *WalkingLab* is the collective walking research-creation practice of Stephanie Springgay and Sarah E. Truman. *WalkingLab* often works in collaboration with other artists and scholars, and the online hub (www.walkinglab.org) archives these networked activities.

The invitation to submit work for this special issue was launched, and a deadline for submission determined, long before COVID-19 became an intimate part of our everyday lives, and as such many of the essays in this special issue do not address the pandemic in a direct way, in the sense that much of the walking research described was undertaken before COVID-19. However, the review and revision process, and the labor of getting this issue to print have been entangled with various lockdown measures, catastrophic global deaths, rising inequalities, and the physical, mental, and emotional toll that the pandemic has caused. In addition, the essays are concerned with feminist, queer, trans, anti-racist, anti-ableist, and anti-colonial walking practices to create a more just future, and as such they are directly and/or tangentially responding to ongoing global crises of place that have existed long before the current pandemic became center stage and eclipsed other pressing issues of place. These crises of place range from climate change and environmentalism, to borders and migration, anti-Asian and anti-Black racism, settler colonialism, internment, displacement, trans rights, gentrification, and urbanization. It is our contention that there is an urgent need for explicit and political attention to critical place research in order for people to understand and develop a critical awareness of how they are connected to, implicated in, and responsible to place. The benefits of critical place research via critical walking methodologies can result in stronger connections and relationships to place, and thus effect greater care, accountability, and advocacy.

## Oblique Agitations of Place

As the essays in this special issue attest, there are a myriad of possibilities for a walking practice, including walking interviews (go-alongs), walking as a form of storying, walking as counter-mapping, walking as citizen science, walking as counter-surveying, walking and speculative fiction, walking tours, walking as a form of refusal and resistance, and walking as a way of being with Country. Instead of inscribing walking methodologies as a set of predetermined procedures, walking becomes an ethical and political accountability and responsibility for how we walk, who walks, and where we walk, or what *WalkingLab* calls critical walking methodologies. Critical walking methodologies account for the ways that walking is imbricated in legacies of settler-colonial harm, white supremacy, and functions to police and regulate bodies (Heddon & Turner, 2012; Springgay & Truman, 2018, 2019; Truman & Springgay, 2019). Furthermore, critical walking methodologies resist inclusive, equal, and depoliticized accounts of walking and movement. As a verb “walking” may be thought to refer to specific kinds of ability and bipedal movement, but disability scholars and activists also use the term when describing their use of mobility devices such as wheelchairs (Chandler et al., 2019; Rose, 2014; Taylor, 2010). As such we take up walking to be responsive of the different ways people move through space, and the different bodies that move, including an attention to pausing, not moving, and not walking (Decter this issue). Syrus Ware (this issue) “crips” walking from a disability justice lens to center disability within walking research specifically attending to the ways disability disrupts “nature” and hiking trails.

Over this past year and a half, we have become acutely aware of walking’s entanglement with ongoing global crises and protest movements. For example, this past month in Canada, in response to more than a thousand bodies of Indigenous children being found in unmarked graves at the sites of former residential schools, people took to the streets to demand greater accountability from the Canadian government and cease naming of institutions after individuals who were key architects of the residential school system. In Toronto, walkers toppled a monument to Egerton Ryerson at Ryerson University, whereas in Winnipeg a statue of Queen Victoria was unseated. Walking enables those advocating for radical change to redefine place, countering dominant and institutionalized narratives of a place. The global Black Lives Matter protests in response to ongoing police violence enacted on Black people in public places and global rallies in support of Palestine further demonstrate the power of walking as a method for calling attention to systemic injustices, and the need for critical engagement with understandings of place.

Those of us engaging with walking methodologies in research need to interrogate how place is entwined with the social, material, cultural, and political dimensions of diverse human bodies, experiences, and communities. Critical walking methodologies understand place as intimately tied to the intersections and flows of race, gender, ableism, capitalism, and settler colonization. The international scholars featured in this issue draw from critical walking research to elaborate on the social, material, cultural, affective, and political dimensions of place. According to Aleut scholar Eve Tuck and settler scholar Marcia McKenzie (2015), place-based research and pedagogy conventionally understands place as a static backdrop devoid of or separate from the social and cultural dimensions of life, is primarily focused on outdoor education in rural spaces, and re-inscribes White settler relations to land (as opposed to centering Indigenous knowledges and decolonization). Furthermore, scholars have argued that to conduct critical place research, we need research methods that break from ableist, racist, extractive, and settler colonial logics and instead focus on ones that are *situated*, *relational*, and *ethical*. We contend that not only do we need empirical critical place research to elaborate on the social, material, cultural, and political dimensions of place; we require critical methodologies to do so: This is where critical walking methodologies become crucial.

Different scholars use different terms to describe critical place research. Tuck and McKenzie (2015) use “critical place inquiry” and argue that it is distinguished from other forms of place-based research because of *explicit and political* attention to place, and it centers Indigenous knowledges of Land. Critical place inquiry, they argue, grapples with place in the context of settler colonialism, globalization, and environmental degradation. The concept of “emplacement” has historically been linked to walking and place-based research (Howes, 2005; Pink, 2009/2015). If embodiment considers the interconnection of mind and body, emplacement understands the interrelationship of mind–body–place. Yet, Tuck and McKenzie (2015) contend that emplacement continues settler-colonial *replacement* of Indigenous peoples and land, as it continues to erase the importance of land and place for Indigenous peoples and their ongoing displacement. As such, they argue for a “deeper consideration of the land itself and its nonhuman inhabitants and characteristics as they determine and manifest place” (p. 40).

The concept of inhabiting place is complicated by feminist environmental scholar Stacy Alaimo (2016) who describes an “ethics-in-place” as a mode of recognizing one’s specific location as entangled within a broad network of more-than human relations. If we are to consider how bodies inhabit places, then we also must recognize the permeability of human bodies with other nonhuman bodies, including places, and as such recognize that transcorporeal ethical dimension of inhabitation.

Attending to the ways that mining and extraction operate through removing resources and chunks of “places” in the service of ongoing imperialism while simultaneously treating particular (racialized) subjects *as* resources, Kathryn Yusoff (2021) links historical and ongoing practices of racial capitalism and exportation logics to the “condemnation of black and brown life across carceral spaces, environmental justice, and toxic atmospherics, as well as the burden bodies have to carry in racialized spaces” (n.p.). This link between underground mines, the dispossession of Indigenous lands, and the shiny (white) surfaces of colonial occupation demonstrate racial logics at work in the production, construction, and maintenance of “place.”

Canadian artist and *WalkingLab* collaborator Camille Turner’s ongoing walking and counter-mapping projects interrogate Canada’s role in transatlantic slavery conjuring up time travelers who have returned to earth to save the planet. In the *Afronautic Research Lab*, they work with citizen researchers to examine the legacies and ongoing implications of slavery. In an iteration of this work in Newfoundland, the focus was on the building of slave ships in the region, the shipping of cod south to feel enslaved blacks, and the fungibility of black bodies with stones. Slave ships built in Newfoundland were sent to Africa ballasted with stones from the Newfoundland shore. The stones were unloaded in Africa and replaced by bodies. Turner’s artistic practice resonates with the scholarship of Tiffany Lethabo King (2019) who conjures the place of the geologic and oceanic formation of shoals (rock formations, reefs, and sandbars)—offshore points of contact and encounter, as well as emergent formations—as an analytic tool that impedes, slows down, and creates a shifting liminal space to navigate Black and Native studies. As a land-sea formation and as a meeting place for Black and Indigenous politics, art, and activism, the shoal becomes a place for imagining future coalitions. King notes that it is often on a shoal that enslaved peoples first disembarked—a point of encounter between land and water: transmaterially both geologic and oceanic. It is for this reason that King finds the shoal an important formation through which to think about Black and Indigenous solidarity and to put in tension the ways that Blackness is always framed as rootlessness and Indigeneity synonymous with territory.

Turner’s performance of a time traveler walking the Newfoundland coast carrying a stone similarly activates what King, citing McKittrick, names as “black Atlantic livingness” (King, 2019, p. 30). The lithic materiality of the stone is intimately tied to people and place.

Furthermore, Black feminist geographer Kathrine McKittrick contends that traditional place research erases Black place-making and as such critical place research must account for Black geographies as sites of contestation and resistance. McKittrick’s (2021) discussions of Black livingness exemplify how through the arts, ritual, and other forms of creative practice Black subjects escape colonial capture and re-making place on their terms and through their creative practices, exemplified by Turner’s and Ware’s (this issue) engagement with Black speculative fiction to build a more just and liveable future.

Putting these place-based theories to work in critical walking methodologies becomes a practice of creating conditions for complex relations, to hold in tension competing or defamiliarizing concepts, and to be “out of place” while simultaneously situated and in relation to place. As Miwon Kwan (2002) wrote about site-specific art, “This means addressing the uneven conditions of adjacencies and distances *between* one thing, one person, one place, one thought, one fragment *next* to another, rather than invoking equivalencies via one thing *after* another” (p. 166). Countering serialization or linear space and time, Kwan insists that place needs to be interrogated as a complex set of relations.

*WalkingLab* attends to such frictional relations through “oblique contours.” This means that the place where the walking event takes place and the concepts activated on the walk need to materialize oblique relations (Truman & Springgay, 2019). We understand oblique as nonnormative, askew, and queer. Furthermore, oblique requires that we consider place as more-than a physical location or what can be seen or experienced in the immediacy. For example, one of the ways we approach this is through what we call a “queer walking tour.” Conventional walking tours can reinforce dominant histories, memories, power relations, and normative or fixed understandings of place. This place-based knowledge can serve various forms of governance or ideology and maintain the status quo, including the ongoing violence of settler colonization and erasure of racialized, gendered, and disabled bodies. Queer walking tours are situated (they happen in a specific location and attend to that place) but simultaneously are “out of place” (meaning that they defamiliarize how diverse publics attune to a place and concepts). One such queer walking tour, *The Bank The Mine The Colony The Crime*, co-organized between *WalkingLab* and Max Haiven, critically examined the financial district in Toronto. This queer walk included lectures and performances on Canada’s legal, financial, and genocidal implication in extractive economies on Indigenous land on Turtle Island and abroad; critiques of the increased privatization of public space; affirmations of the need for harm reduction in the city; descriptions of the experiences of international students in the market economy of higher education; interventions from global mining activists; and expositions of how gentrification has affected Toronto’s LGBTQ+ (lesbian, gay, bisexual, transgender, queer or questioning) community and Church Street. Thinking and walking with oblique contours become a way of exposing and defamiliarizing a place—in this example Toronto’s financial district—and the many more-than-human entanglements that sit in tension with a particular place. The essays in this special issue not only elaborate different walking practices such as counter-mapping, walking interviews, and so on; they simultaneously *agitate* place as *oblique* social, material, and political relations.

## Walking in Times of Ongoing Global Crises: The Essays in This Special Issue

The first two essays in the special issue examine National trail systems and their political implications in the making of nations and citizenship. Toby Beauchamp walks the Arizona Trail (AZT) as a case study to consider the ways that hiking is made to serve a political purpose in part through its representation as being an apolitical activity. Beauchamp analyzes the discursive ways that opposition to the border wall between the United States and Mexico employs the figure of the hiker as one that is both beyond the realm of politics and aligned with practices of good citizenship. In contrast to this figure of the apolitical hiker, the migrant is often positioned as undertaking a criminal form of walking that the wall is meant to prevent. Nationalist discourses that inscribe the Arizona Trail as pristine and scenic only further materialize border militarization. Beauchamp argues that studying the relationship between the AZT and border militarization, we can better understand not only how politics structure hiking but also the political effects of insisting that hiking is not political.

Canadian artist and walking scholar Leah Decter discusses her Critical White Settler Projects on the Trans Canada Trail (TCT) as artistic and methodological responses to the imperative for non-Indigenous people (BPOC and white settler) to do our part to shift colonial norms instead of putting this responsibility and labor on Indigenous peoples. Using a walking research-creation project as an exemplification, Decter highlights how critical white settler projects—when operating in conversation with Indigenous methods as opposed to appropriating or overshadowing them—might contribute to specific and overarching goals of decolonization.

Waka Waka and Gooreng Gooreng scholar Sandra Phillips’s article foregrounds how walking is inherent to the experience of being Aboriginal: for millennia, along songlines, trade routes, and in place. Phillips reflects on episodes during of her life on Country, in the academy, and in different urban places across the land known as Australia, where walking as an Aboriginal woman calls up intergenerational memories.

Sam Steigler’s essay utilizes the method of the walking go-along interview on a series of walks undertaken by a cis White queer man and a Black trans young woman, along the Christopher Street Pier in the West Village of New York City. The narrative form of the interview works against White-cis and hetero-normative senses of time-keeping and place-making by illuminating how bodies and social positions were perceived in relationship to each other and the environs of the go-along. The interviews and the researcher’s reflective narratives account for the ways that the Pier has long been an important public and community space for trans and queer Black and Latinx communities, especially young people, and the processes of gentrification that have made this place inhospitable to these communities.

Brazilian scholars César Martinez and Gabriela Gois adopt the concept of *intersectionality* to construct an analysis that considers the effects of the colonialism, racism, and sexism in walking scholarship in a Latin American context. Analyzing the idea of *risk*—when some subjects are identified as “not belonging” in space—they argue that the interactions between *gender*, *race*, and *place* impose a local condition on the knowledge that can be produced by Afro-Latin American walking researchers.

Accounting for the ways that the act of walking and place are sites of marginalization and hostility, Taien Ng-Chan develops what she calls a method of marginal walking as a critically creative framework that nourishes and supports the spaces of the margins in response to everyday racism—specifically anti-Asian racism that has risen during the pandemic. Ng-Chan investigates how news of racist incidents circulates through digital networks that are entangled with quotidian places such as parks, grocery stores, and public transit. Through such concepts as auto-cartography and strata-mapping as critical walking practices, she examines how conscious acts of sensing and intervening in marginal everyday space can contribute to the creation of alternative narratives and knowledge that is necessary for change.

Drawing from her ongoing research into citizen science and the more-than-human, Jennifer Gabrys’s essay asks, “How does the forest walk?” She considers “forest-based vectors of movement” in relation to existing critical walking scholarship and investigates how digital technologies (platforms and apps) co-constitute forests as distributed sites that travel. Gabrys argues that being attentive to different modes of forest inhabitations and the movement of forests—including more-than-human inhabitants and different ways of knowing—is crucial in a time of climate change, ongoing settler colonialism, and extractivism.

Incorporating digital technology, drawing, and walking Syrus Ware’s essay explores the concept of forest bathing and a “cripped” walking pedagogy immersed in a disability justice lens as a strategy for revolutionary prep and community building with youth from an urban center. Mixing personal reflections from childhood encounters with nature, Ware weaves stories from a day-long artistic exploration on the Bruce Trail, in Ontario, on Northern Turtle Island with youth from T’karon:to (Toronto). This article considers the radical possibilities of sharing drawing practice and speculative fiction, with environmental knowledge to ensure the next generation survives any future changes or revolutions. Ware’s essay further complicates Beauchamp and Decter’s essays on National Trail systems, as the Bruce Trail is a 975 km trail in Ontario held in conservancy by settlers. Walking with BPOC youth Ware highlights the ways that walking can refuse totalizing narratives and become a means to imagine a future otherwise.

Continuing to entangle storying and walking, Siddique Motala and Vivienne Bozalek’s essay is situated in District Six, a well-known site of apartheid forced removals in Cape Town South Africa. Using walking and a diffractive cartographic methodology, they map the site by means of geographic information systems (GISs) and oral history with intergenerational families displaced from this place during apartheid. Extending counter-mapping work, the authors explicate a practice they call counter-surveying that recognizes the hauntological power of walking, attending to the ghosts of District Six while interrogating issues of land and education, and opening up a space for Otherness.

The practice of storying and counter-mapping is further explored in Kimberly Powell’s essay on redlining and incarceration in Japantown, San Jose, California. Drawing on assemblage theory, Powell examines spatial racism and its affective contours in the history of and present-day lived experiences of Japantown. Using a walking interview method she calls StoryWalks, Powell is attentive to the ways that movement might breach the assemblage and, in doing so, reach toward radical reformation through memorialization, community activism, and development.

Renaissance scholar James O’Neill and sociologist Maggie O’Neill focus on a “constellational” walk they completed in the Boboli gardens in the footsteps of Poliphilo in Italy as a method for interdisciplinary collaboration. The Boboli gardens are a physical place inspired by the narrative of the Renaissance text *Hypnerotomachia Poliphili*. Through walking physically through the gardens and discussing episodes in the text, O’Neill and O’Neill exemplify the transdisciplinary potential of walking methods for critically engaging with history, literature, and place.

The essays in this special issue demonstrate the various ways that critical walking methodologies attend to the complex and oblique contours of place. In the midst of ongoing injustice and global crises, walking, we contend, enables researchers and participants to engage in critical and creative practices that are in situ and responsive to place. Beyond simple pedestrianism or the movement of going from one point to another, critical walking methodologies are accountable to the different ways that different bodies and people move, and sociopolitical and geological inheritances and affects of the places where one walks. Attending to oblique contours and relations of place demands that we consider more-than the physicality of a location to defamiliarize and agitate place-based relations.

As the essays articulate, walking is never neutral, rather highly politicized. But so too, as the various authors contest, walking has the potential to create more just and liveable worlds. *WalkingLab* and the authors we have gathered in this issue continue to practice walking-with. Walking-with is a deliberate strategy of unlearning, unsettling, and queering walking. To articulate walking-with, we have previously drawn on the work of Indigenous scholars Juanita Sundburg, Jon Johnson, and Bonnie Freeman. *With* is a preposition. It is used to indicate associations and connections between entities. However, walking-with is more than merely additive (it is not a + sign); it is the ethico-political *(in)tensions* (Springgay & Truman, 2018) brought to bear on walking, the place where one walks, and the concepts, bodies, and archives that are co-composed through walking. What this means is that *with* does not simply indicate that a walker is walking-with a dog, with a lake, or with another walker. Rather, with is a *milieu*—an active set of relations that are composed of dimensions and vibrations that materialize a moment of space-time (Truman, 2021).

*WalkingLab* would like to thank the reviewers who generously gave their time and labor during a challenging year, and to the authors for their careful and attentive walking-with practices.
